# Barriers and facilitators of opioid treatment among Indigenous Syringe Services Program clients

**DOI:** 10.1186/s13722-025-00604-8

**Published:** 2025-10-16

**Authors:** Jordan Stipek, Jennifer J. Mootz, Frank L. Johnson, Kevin A. Hallgren, Atasha L. Brown, Alexandra Perron, Aanji’bide Community Action Board, Clinton Alexander, Brenna L. Greenfield

**Affiliations:** 1 University of Minnesota Medical School, Duluth Campus, 1035 University Drive, Duluth, MN 55812 USA; 2 Department of Psychiatry, Columbia University, 1051 Riverside Dr, New York, NY 10032 USA; 3 Department of Psychiatry and Behavioral Sciences, University of Washington, 1959 NE Pacific Street, Seattle, WA 98195 USA; 4 Tribal Nation Partner, St. Paul, MN USA; 5 Present Address: Department of Emergency Medicine, Regions Hospital, 640 Jackson Street, St. Paul, MN 55101 USA; 6 Present Address: Department of Emergency Medicine, Jackson Health System, 1611 NW 12 Ave, Miami, FL 33136 USA

## Abstract

**Introduction:**

American Indian and Alaska Native individuals are disproportionately impacted by the opioid epidemic, partially due to structural racism. Tribal nations and communities are finding innovative ways to provide opioid use disorder (OUD) treatment, but barriers to medications for opioid use disorder (MOUD) remain. This study surveyed Indigenous clients at a Syringe Services Program about barriers and facilitators to OUD treatment.

**Methods:**

Interviews were conducted with 27 Indigenous individuals who had used opioids in the past month and were receiving opioid harm reduction services from a tribally-run Anishinaabe Syringe Services Program (rural Minnesota). Participants were asked five questions in interview style format about their experiences with opioid use disorder care with a focus on barriers and facilitators. The coding team analyzed interviews utilizing the Collaborative Story Analysis method to highlight overall impressions of participants’ narratives.

**Results:**

There were 27 participants: 48% male and 52% female. The main themes of barriers and facilitators were connection to others, flexibility of treatment services, and ensuring individual needs were met. Having a positive relationship with providers (e.g. non-judgmental), access to MOUD and Harm Reduction services, and minimizing assessment requirements prior to starting treatment were some of the most frequently identified facilitators to care. Lack of transportation, prioritizing care for others, and turbulent relationships with providers and certain aspects of care services were identified as barriers.

**Conclusions:**

Study participants cited clear barriers and facilitators to accessing OUD treatment in a rural Anishinaabe Tribal Nation in Minnesota. The Tribal Nation has already implemented several strategies to improve access to MOUD care (e.g., hiring additional drivers to help with transportation, facilitating immediate MOUD care prior to an intake, if needed, and giving take home MOUD doses). Tailoring services to address identified barriers and leverage facilitators of connection and flexibility will enhance care.

**Supplementary Information:**

The online version contains supplementary material available at 10.1186/s13722-025-00604-8.

## Land acknowledgement

Aanji’bide (Changing our Paths) research collective acknowledges that this work was conducted on the traditional, ancestral, and contemporary lands of Indigenous people. The Aanji’bide research collective acknowledges that this land is home to the Ojibwe people, the Dakota and Northern Cheyenne people before them, and other Native peoples from time immemorial. We recognize the land in this territory and beyond holds historical, spiritual, and personal significance for many Native people, and we affirm the sovereignty of Tribal Nations (adapted from University of Minnesota Duluth [38].

We realize that, in 2025, land acknowledgments are best paired with action plans [39]. Thus, examples of how Aanji’bide acts to support Tribal Sovereignty and survivance include: (1) centering Anishinaabe and Indigenous culture, wisdom, and knowledge; (2) paid co-creation roles for Anishinaabe and Indigenous knowledge holders and Community Members on the team; (3) continued research capacity building for Tribal Nation Community Members via training and mentoring in research ethics, research design and analysis, presenting at scientific conferences, and writing research publications; (4) framing statistics on opioid overdose inequities in their context and roots (e.g., resource and land theft, boarding schools); and (5) training and including non-Native allies as research partners, with a focus on cultural humility.

## Introduction

Opioid overdose deaths in the United States continue to be a major public health crisis . Although there is variation from state-to-state, in 2022 opioid overdose mortality rates for American Indians and Alaska Natives (AI/ANs) were higher than any other group in the United States (Opioid Overdose Prevention in Tribal Communities). In 2021, American Indians in Minnesota had 10 times greater risk of dying due to opioid overdose compared to white Minnesotans [32]. This disproportionate impact of the opioid crisis on American Indians and tribal communities is due in part to the collective impact of unaddressed historical traumas, structural racism, and inadequate policies related to AI/AN health (e.g., inadequate funding for Indian Health Service, despite treaty obligations to fund American Indian healthcare) [4, 23, 24, 26].

Medications for opioid use disorder (MOUD), particularly buprenorphine and methadone, are effective treatments that decrease the risk of opioid overdose and mortality [8] [25] and are a vital strategy to address the opioid overdose epidemic. However, MOUD access and effectiveness is limited by barriers to MOUD treatment initiation and retention [3]. For example, epidemiological studies suggest that in 2022, only 25% of people with OUD received MOUD [33]. Emergency departments (EDs) are one of the primary medical settings that people with OUD interact with, and thus are a principal opportunity for engaging people in opioid treatment. A scoping review by Bozinoff et al. [5] found that main limitations, at least in the ED setting, to initiating buprenorphine for MOUD include failure to address structural stigma, high client complexity, and an increasingly toxic drug supply (namely fentanyl). However, the authors also cited an “urgent need for research that explores service user perspectives” with a hope of increasing MOUD initiation in the ED. Thus, there is a critical need to understand barriers and facilitators to MOUD treatment, especially as reported by individuals with OUD who are not yet engaged in MOUD treatment.

American Indians often face significant barriers to accessing MOUD [14, 22, 40]. Research suggests that barriers to MOUD among American Indians are systemic, linked to colonization, and are connected to social determinants of health—conditions within a home, family, school, and community that can impact a person’s ability to be healthy. These barriers include: (1) limited integration of MOUD into American Indian-serving clinical settings (including due to structural and socioeconomic factors; [14, 21, 27], (2) concerns about integrating MOUD with American Indian holistic and traditional healing models, (3) treatment access barriers associated with rurality [18], and (4) a lack of culturally-relevant research with American Indians on the topic [14, 21, 27].

At the same time, many American Indian and Alaska Native Tribal Nations have taken thoughtful steps to support people who use opioids and mitigate barriers to OUD services. These have included, for example, implementing harm reduction services, peer support, and comprehensive treatment programs that are informed by Indigenous values and aimed at supporting health and wellness of Indigenous people who use opioids [34] [19]. These programs often focus on the importance of relationships (with other people, with the community, and with traditional culture) in healing and have included approaches focused specifically on strengthening these relationships. In addition, the sovereign status of Tribal Nations creates unique opportunities to innovate MOUD programming and approach care from a holistic perspective.

One qualitative study with a national sample of AI/AN people who inject drugs (n = 32) identified barriers to treatment and harm reduction services including stigma, variability in readiness for treatment, and lack of awareness or access to of MOUD, harm reduction, and psychosocial recovery services [17]. However, to our awareness, much of the remaining research on barriers and facilitators to MOUD faced by AI/ANs has focused on the perspectives offered by clinicians, healthcare administrators, and public health experts [22, 27, 40], and less is known about the specific barriers to MOUD treatment as experienced by AI/ANs using opioids.

### The present study

This study is part of Aanji’bide (Changing our Paths), a NIH-funded research partnership looking at the OUD Cascade of Care in partnership with an Anishinaabe (Ojibwe) Tribal Nation in rural Minnesota (NIDA R61/R33DA049386). Community-based participatory research undergirds the research processes: The topic of study is important to the Tribal Nation and Tribal Nation Community Members are co-investigators in the research. The team includes an 11-member Community Action Board composed of Community Members with lived experience with opioid use, family members of people who use opioids, individuals with leadership roles in the Tribal Nation, and individuals who provide treatment and outreach services for people using opioids. The Aanji’bide Community Action Board meets about once a month and guides the research focus and design, data collection, data analysis, and dissemination. In addition, the Community Action Board takes what is learned in the research and uses it to inform Tribal Nation opioid services in real-time.

To address the gaps in the literature, the current study aimed to understand barriers and facilitators to opioid treatment for American Indians from the perspective of people using opioids. To achieve this aim, we interviewed Indigenous individuals who had used opioids in the past month and were receiving services from a tribally-run Syringe Services Program that offered syringe exchange and other harm reduction services (clinic description in Methods section).

**Fig. 1:**
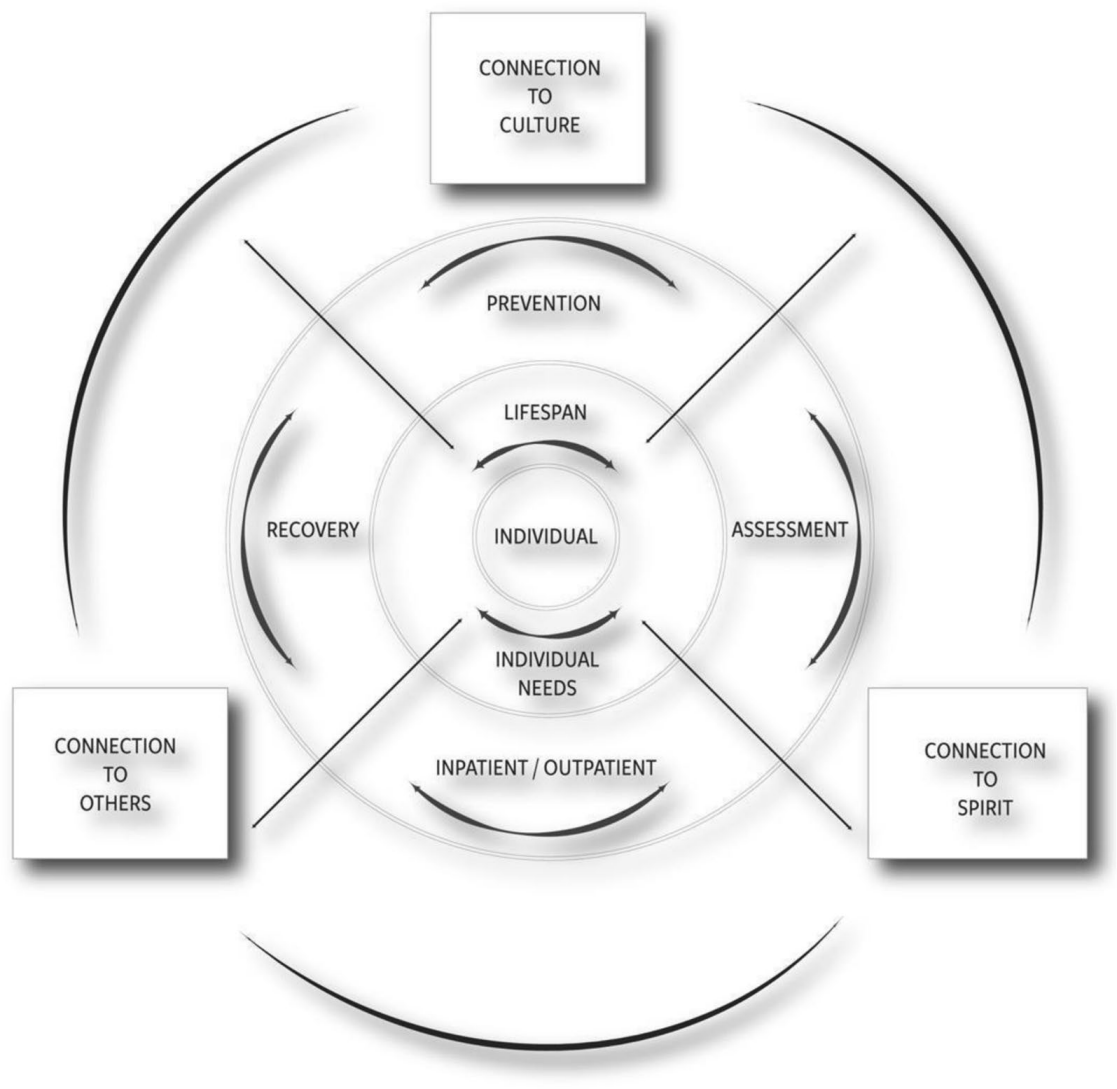
Aanji’bide (Changing our Paths) model of opioid recovery and change [12]

We framed our results around the recently developed Aanji’bide Model of Opioid Recovery and Change [12] (Fig. 1)—an OUD public health framework that was collaboratively developed by this research team. The model offers an alternative to the OUD Cascade of Care public health model [29] and highlights resilience through connection to culture/spirituality, community, and others. While the Cascade of Care provides a useful, population-level snapshot of engagement with services along a linear continuum, from diagnosis to treatment to recovery, it is rooted in a Western biomedical model that may not best represent Indigenous worldviews. In contrast, the Aanji’bide model is non-linear, culturally grounded, and emphasizes cyclical pathways of recovery. It acknowledges spiritual and community connection as key facilitators of healing and recognizes that recovery journeys are multidirectional and deeply shaped by social determinants and cultural connectivity. This approach attempts to center Indigenous cultural wisdom and community voice, and highlight a more accurate and respectful framework for interpreting treatment engagement and barriers in tribal contexts. To our awareness, no studies have examined barriers or facilitators to OUD treatment through the Aanji’bide Model of Opioid Recovery and Change, and prior studies that draw on the OUD Cascade of Care public health model often have overlooked the perspectives of people with OUD who are engaged in harm reduction services. Through our findings, we aim to shed light on factors that impact the ability for American Indians engaged in harm reduction services to receive OUD treatment, doing so with humility and attention toward strengths-oriented actions that could be implemented to make MOUD access easier.

## Material and methods

### Study setting: Tribal Nation OUD treatment services

#### Syringe Services Program

The Syringe Services Program is staffed by public health nurses and rotates between four locations on the reservation so that services are more accessible to clients on this geographically dispersed Tribal Nation. The program offers sterile needles and equipment, health education, STI testing, vaccinations, and naloxone. Some mobile services are also provided. Staff assist clients with access to treatment services and help them navigate through programs to assist with housing, emergency assistance, and medical care. They partner with other Tribal Nation programs to provide clients with meals and snacks, and to provide baseline medical assessments and treatments to catch health concerns before they become urgent.

#### Other OUD services

In March of 2022, at the time of the research interviews, the Tribal Nation offered substance use disorder treatment services including buprenorphine for MOUD. If individuals wanted funding from the State of Minnesota for their SUD treatment (i.e., they did not have private insurance), they needed to complete a “Rule 25” assessment; nothing could be done without it. This four or five-hour long assessment required an appointment and then it would take a couple more days before treatment could begin (same-day treatment was not available). On June 30, 2022, the state of Minnesota replaced the Rule 25 assessment with a comprehensive assessment, with the intention to make treatment access easier. The comprehensive assessment can be done within an hour, and treatment can be started before the assessment is completed (although the assessment is still mandatory). Prior to the introduction of the comprehensive assessment, the Tribal Nation had begun experimenting with walk-in Rule 25 assessments (versus scheduled) to reduce barriers for clients.

Cultural services for individuals with opioid use disorder were available in the community and in some treatment programs (e.g., sweat lodge). Community outreach to people using opioids included naloxone distribution and community events with information about OUD treatment programs. Methadone was only available from non-Tribal programs in an urban area an hour or more drive away. The region also has an American Indian/Alaska Native-specific residential treatment center, and other residential treatment centers. Movement between the reservation, neighboring cities, and the Minneapolis/St. Paul area is common. There were additional buprenorphine providers in towns and cities bordering the reservation, either independently operated or affiliated with large healthcare systems.

### Participant eligibility criteria and recruitment

To be eligible for participation, individuals needed to: (1) be 18 years and older, (2) self-identify as Indigenous, (3) be established clients at the Tribal Nation Syringe Services Program, (4) have used non-prescribed opioids in the past 30 days, and (5) have capacity and willingness to provide informed consent. Participants were told about the study exclusively by clinic staff, who are trusted by their clients. A week or two in advance of the interview dates, staff informed clients about the optional opportunity by offering them a copy of an informational flyer about the upcoming interviews. On the day of the interviews, staff informed clients that the interviews were happening, and to speak with the on-site research team member if they would like to learn more. Interviews occurred in the Tribal Nation Syringe Services Program. The researcher reviewed eligibility criteria with each person. If they met eligibility criteria, the researcher reviewed the consent form and then asked for verbal consent to participate. This was a convenience sample of clients who came into the clinic on the interview days.

### Measures

#### Interview guide

The Interview Guide (Appendix) was developed collaboratively by the Aanji’bide Community Action Board (CAB) and authors BG and JM. In an early meeting, the CAB asked that two CAB members with lived experience using opioids take the lead in developing questions. Thus, several meetings with these two individuals resulted in a draft Interview Guide that was brought for final review to the full CAB. The full process included six drafts generated via discussion and meetings between June 2021 and November 2021. For the interview guide, we emphasized that there were no right or wrong answers, and that the interviewers were there to learn from participants. We wanted to approach the work from a place of cultural humility, recognizing that “no one is better than another” (p. 423; Elder Noreen McAteer; [13]).

Interview questions covered the topics of (1) barriers and facilitators to OUD treatment, (2) mental and physical health, (3) connection to traditional Anishinaabe culture and spirituality or other religious beliefs, and (4) self-care. This paper focuses specifically on Topic 1: barriers and facilitators to opioid treatment. The two questions about barriers and facilitators to opioid treatment were:

1. What things have made it easier for you to access opioid treatment services?
2. What things have made it more difficult for you to access opioid treatment services?

When barriers and facilitators were mentioned outside of these two specific questions, they were included in the coding process and are discussed in this paper.

#### Demographics questionnaire

A brief demographics questionnaire, completed via self-report on a tablet, asked about age, gender identity [31], whether the individual had ever taken buprenorphine (Suboxone) prescribed to them by a doctor or health care worker, and whether they had ever gone to a Tribal Nation substance use disorder treatment program.

### Procedures

#### IRB approval

This study was approved by the Tribal Nation IRB (12/28/21) and the University of Minnesota IRB (STUDY00013546). The IRBs approved a waiver of written documentation of consent for this study because of its sensitive nature. All participants provided verbal consent for participation.

#### Interviews

For those who gave verbal consent, an audio recorder was turned on, and the researcher went through the Interview Guide (Appendix). One person did not wish to be audio recorded, and their responses were written down by the researcher instead. Following the interview, the audio recorder was turned off, and participants completed the Demographics Questionnaire in Qualtrics on an ipad. They received a $20 Visa gift card and a gift of tea and cedar salve made by a local holistic practitioner and Community Action Board member.

All interviews were conducted in March of 2022 by BG, a clinical psychologist and assistant professor at a medical school. Interviews were conducted over a two week period. Each of the four Syringe Services Program locations was visited once. The number of participants at each site ranged from three to 14 individuals. Individuals could participate on their own or in a group of their choosing. No additional parties were present aside from the participant(s) and interviewer. Seventeen interviews were with a single participant and five interviews were in pairs, for a total of 22 interviews and 27 participants overall.

Given that individuals were visiting the Syringe Services Program for services and expecting to be in and out quickly, we kept interviews brief. The average interview length was eight minutes (ranging from three to 15 min). Every interview followed a standard, IRB approved guide that began with an introduction by BG, who stated her credentials and her affiliation with the University. BG also ensured that part of the introduction included stating the goal of the interviews (i.e. “I am here to learn from you”) and ensured participants were able to ask questions prior to the start of the interview. We did not track rates of non-participation. Most individuals who came into the Syringe Services Program the day of the interviews and met eligibility criteria did participate. A handful did not participate for lack of time. No repeat interviews were conducted.

### Data analysis

Audio recordings of the interviews were transcribed by a professional transcription service. Both the raw audio files and the subsequent transcribed interviews were stored on an up-to-date and secure institutional server (Box). Transcripts were not returned to participants for comment or correction after the interview and transcription process. We did not maintain personal identifiers to protect participant confidentiality.

We initially planned to use thematic analysis to interpret qualitative data from the transcripts. Thematic analysis breaks down stories into separate fragments. However, the qualitative analysis team discussed this method and felt it did not honor their stories and that the use of discrete codes detracted from the context and provided a less holistic perspective. The team explored use of Indigenous-aligned analysis methods, and ultimately used an adapted Collaborative Story Analysis Method modeled after “*What Touched Your Heart? Collaborative Story Analysis Emerging from an Apsáalooke Cultural Context*.” [11]. Details of how the Aanji’bide Research Team used Collaborative Story Analysis are provided in [41]. Collaborative Story Analysis builds on standard methods of rigor in qualitative research, such as analysts’ theoretical triangulation and being inclusive of multiple viewpoints and professional backgrounds (e.g., lived experience with opioids).

Using the Collaborative Story Analysis Method as a guide, our qualitative analysis team of 4–6 members met weekly for 90 min to discuss each transcript as a group over a five month period. A member of the team would display a transcript using the screen share feature on the virtual platform. The team would read the transcript silently and then discuss page by page. Discussion was guided using the following questions to prompt conversation: “What stands out to you,” “what feels important,” and “what do you notice.” Priority was given to Community Members in discussion. After completing a transcript, the team would reflect on the transcript as a whole and give their overall impressions of the important aspects of the client’s narrative.

Once the analysis team read and discussed all the transcripts, they had an in-person gathering near the Tribal Nation to reflect on the analysis process and overarching topics that appeared across transcripts. They presented the main topics to the Syringe Services Program staff and the CAB to elicit input, feedback, and recommendations for practice. Following this in-person meeting, the team resumed meeting virtually. Notes were organized according to the central research questions (i.e., barriers and facilitators to MOUD care) such that all team discussion related to a research question was placed in that category. The remaining virtual meetings were dedicated to reviewing the notes of the group discussions and reflecting on stories’ messages related to research questions and how those messages were shared across participants. A de-identified summary of the data was generated but this was not shared with individual participants for feedback. We asked for feedback from clinic staff and the Aanji'bide CAB.

## Results

### Participant description

The study included 27 participants: 48% identified as male and 52% as female. All identified as Indigenous. Participant age ranged from 24 to 60 years, with an average age of 39 years. Seventy percent (*n* = 19) had gone to a Tribal Nation substance use treatment program at some point. Fifty-nine percent (*n* = 16) had ever been started on buprenorphine by a doctor or health care worker.

### Qualitative findings

Study participants noted several barriers and facilitators of care. At times they referenced MOUD services and other times they discussed what they liked or disliked about Syringe Services Program services. Three primary themes of barriers and facilitators to MOUD services arose. These themes were: (1) connection to others; (2) flexibility of services; and (3) social determinant needs as barriers to treatment. The Aanji’bide Model of Opioid Recovery and Change (Fig. 1) was used to frame these findings.

### Theme 1: connection to others

Related to the outer circle of the Aanji’bide Model of Change, participants identified several barriers and facilitators regarding connection to family, providers, and the community (Fig. 1). Participants across interviews discussed the importance of connections. Family members were a prominent facilitator of treatment seeking. For example, one participant (#21) noted that “just thinking of the grandkids, my kids’ life” helped them to seek treatment. A minority of clients described how caretaking responsibilities limited treatment options, especially if options were inpatient residential programs and/or they were services located outside of the tribal nation. For example, one participant (#12) shared, “My mom just like she has cancer or whatever, and she fell down… and broke her leg. So, I had to put a pause on a lot of stuff to go take care of her.” Typically, however, connections with family members were noted as facilitators for accessing care.

What has made it easier? I don’t know, I have a family member that’s in like, chemical dependency. So, she helps me out with a lot of stuff too. And now I’m not on opioids no more because I went to jail for a couple months and got out and just haven’t gone back to them. (Participant #7)

A recommendation regarding family was to provide options for childcare or other activities for logistical reasons related to childcare responsibilities and so family members could remain connected during treatment.

Respondents also stressed the importance of connection with providers. While they did not explicitly state that connection to providers was a facilitator, discussion centered around relatability with providers and how providers could effectively communicate with clients. The following excerpt, for example, highlights the importance of the provider–client connection, with the participant recounting an experience when they advocated heavily to be assigned a provider with whom they could connect. This advocacy included seeking services in another village to ensure that connection.

Well, I’ve been in and out of treatment I don’t know how many times over the years and every treatment that I’ve been into, I’ve always had a counselor where they’ve had a prior addiction so they can relate to a lot of what I’m saying, and I can relate to what they’re trying to tell me. There’s been one treatment center that I don’t think is open anymore, it was up in [nearby inpatient treatment center, non-tribal], that’s the only time I’ve ever had a counselor that was book smart, book educated on addiction. And I could tell from the second I walked through the door. And I tried to get a different counselor, and the director wouldn't let me do it and he’s asking me why. And I said because this lady is fucking, she’s book smart, she’s never had an addiction in her life. I need somebody that’s been down these roads. I need somebody that I can relate to. And yeah so he still wouldn’t let me change my counselor so I ended up taking off from treatment and I got myself into a different place up in [name of neighboring Tribal Nnation] which went a lot smoother but once again, if people don’t have other people out there that can at least relate to what they’re going through on a certain scale, they’re not going to try to take that first step to try to get the treatment. They’re worried about a lot of shit the way it is. (Participant #8)

Study participants expressed a preference for communication that was nonjudgmental and accepting, a style that supports the client-provider relationship and enhances rapport. Some perceived a nonjudgmental communication style as present in the Syringe Services Program and endorsed this approach.

I would have to say...what has made it easier? Definitely [the Syringe Services Program] has made it a lot easier. A lot easier on my anxiety, nerves, questions, all that. The ladies through [Syringe Services Program] are very valuable to us as an addict right now. Not only will they...I don't know, shovel out the path to sobriety if that’s what we needed or whatever the case may be. They’re always there. They’re there to keep us safe, keep us with clean tools to do our business or whatever. I don't know. (Participant #1)

Others gave examples of communication that impeded connection between the provider and client and was a deterrent for service-seeking.

I think there’s a lot of misconception about addicts and stuff and a lot of the people, a lot of the times working in the CD [chemical dependency] offices, aren’t addicts or they don’t understand what some of these people are going through. So, and some of these people walk in and these guys have attitudes or talk to people a certain way. It can rub a person the wrong way and get them to change their mind about going to treatment or doing something. (Participant #8)

Finally, considering connection to others in the community, some clients described attending MOUD services and re-entering the community and then being around others using opioids as a barrier.

### Theme 2: flexibility of services

An additional concept that interviewees discussed was their relationship with existing treatment services, which maps onto the inner circle of the Aanji'bide (Changing our Paths) Model of Opioid Recovery and Change (see Fig. 1). Participants cited both barriers and facilitators in this regard. Participants emphasized important facilitators related to flexibility in scheduling that included walk-in Rule 25’s and access to MOUD and Harm Reduction providers without having a prior appointment scheduled. As described above, the Rule 25 assessment was required to receive public payment for SUD services. At the time of these interviews, the Tribal Nation had begun offering walk-in versus pre-scheduled Rule 25 assessments. Participant #4 shared that walk-in Rule 25’s are “easier to get to I guess or not having to have a set appointment because I can never follow appointments or a set schedule.” Another participant shared how this flexibility helped prevent them from relapsing.

It was easier for me to get going. Like I wouldn't have to, like okay if I was going through withdrawals like I did the last time I was on them, I went through withdrawals and oh you got to go do a Rule 25 then we got to wait for that. It’s like I’m trying to get help and you guys are making it go up farther and farther. Pushing me up farther, so I relapsed. I went back to using, I didn't even start. So now I just went up there and I started right away. And it helps. It helps a lot. (Participant #16)

I guess it could be pretty easy to go into treatment or go ask for help. We could walk right in up at the [tribal MOUD program]. They take in walk-ins. I have family that works in substance abuse so, you know, it wouldn’t be hard for us to get help from anyone, but I just choose not to. (Participant #15)

When trying to meet scheduling requirements, some participants also cited organizational scheduling factors that they perceived as hindering access to existing care. Specifically, participants shared difficulty scheduling appointments or getting return calls from treatment services. Participant #10 shared that “I've been trying to call [treatment service center] for maybe the past three months, and she is not returning any of my calls. I've talked to [name] supervisor and somebody else was supposed to call me, and they never called me. So it's frustrating because I've been trying to get into treatment, and it's tough to quit on your own. Around here you just can't do it.”

Other sentiments related to MOUD care were preferences for flexible options regarding available medications and services. Two participants noted that they would like to take methadone, but that it was not easily available. One said:

I want to do methadone. I don’t want to have to wean off of suboxone. Coming down is really bad. Takes everyone years to get off of it. For methadone, transportation is an issue. [Off-reservation city 1] is closer, but the cost is high. [Off-reservation city 2] has a free program. (Participant #6)

Clients liked opportunities for syringe exchange. Another example of flexibility as a facilitator of opioid treatment was being able to receive MOUD immediately, an option many participants liked. Other examples were having the option of inpatient services and flexibility regarding dosing (i.e., allowing clients to schedule dosing).

### Theme 3: unmet social determinants of health as barriers to treatment

A third major theme was the focus on individual needs grounded in social determinants, as is demonstrated in the center of the Aanji’bide Model of Opioid Recovery and Change (Fig. 1). Recognized in terms of both barriers and facilitators, participants’ discussion highlighted examples of social determinants of health as necessary for being able to seek and attend treatment. A determinant highlighted across almost all interviews was transportation, both public and private, as a major factor affecting ability to access care services. In most cases, participants described transportation as a barrier given they did not have access to their own transportation (i.e., cars), and treatment programs may not have had enough transportation options to assist participants. Public transportation options, such as buses, vans, or rideshare services, were mostly unavailable given the rural setting.

Transportation is a big thing. And then, or like there’s a homeless program here and there’s a lot of people that are homeless like I said you can’t get a house if you don’t have your kids, you can’t have your kids if you don’t have a house. It’s like a double conundrum or whatever the hell they call it. (Participant #16)

Perhaps given needs related to access to transportation, participants expressed frustration with MOUD treatment demands that required frequent office appointments (e.g., for frequent urinalysis testing), and other logistical barriers that were perceived as cumbersome for accessing care.

Additionally, some participants shared that legal issues were a barrier to accessing treatment.

... Well, my car got taken because I didn't have a license and now I’m here, no transportation, so that screws up my work conditions. I was trying to get a job at a casino. Just getting back and forth because they got one transport vehicle that comes and gets you to go [to the program]. And it's just, you can't do anything around here without transportation. (Participant #16)

Participants cited lack of MOUD options while detained, inability to fill out Rule 25 forms or intake assessments while detained, and being discharged from treatment programs because they were arrested or were otherwise involved in legal troubles. One individual also described that their previous convictions for a DUI prevented them from keeping their drivers license, which subsequently hindered their ability to drive to appointments regarding their substance use. They also described compounding barriers to independent transportation, such as needing to complete treatment, get multiple letters of recommendations, and pay other fines. Recommendations to improve social determinants were to improve socioeconomic stability by MOUD programs providing employment and housing opportunities.

## Discussion

The 27 Indigenous research participants who were accessing a Syringe Services Program in a rural tribal Nation in Minnesota had clear ideas on what would impede or support seeking MOUD treatment. Participants described several themes that mapped onto various levels of the circular Aanji’bide Model of Opioid Recovery and Change. These themes focused on connection to others, flexibility of services, and needs that are grounded in social determinants and serve as barriers to seeking and receiving care. To our knowledge, this is the first study done with Indigenous participants who were accessing a Syringe Services Program to learn their perspectives on barriers and facilitators to opioid treatment.

### Theme 1: connection to others

One of the central findings was that connection served as an important facilitator for seeking care. The topic of connection arose in several ways: connection to others (usually family), connection to providers, and connection to community. Consistent with what participants shared about connection to family as a motivator for seeking treatment, a systematic review of five randomized controlled trials on the relation between social support networks and improved MOUD treatment outcomes found that among partner, family, and peer networks, the family social support network had a particularly positive influence [15]. An analysis of responses among American Indian youth (n = 19,067) from the New Mexico 2000–2019 Youth Risk Resiliency Survey established that reporting more social support was associated with lower opioid use [1]. This study also showed that for youth who were using opioids, social support was associated with lower odds of having made a past suicide attempt.

Participants also indicated that connection to providers facilitated care [2]. They emphasized the benefits of communication that was nonjudgmental and accepting. The importance of exhibiting a nonjudgmental attitude when working with people living with stigmatized behaviors, such as opioid use, is well established in the literature as an important mechanism for delivering effective therapeutic care. Many of the findings in this study can resonate with people with OUD as a whole, not just for AI/AN individuals. Being accepting of where people are in terms of readiness to change is a core component of motivational interviewing, an evidence-based therapy for multiple forms of substance use and other behaviors, for example [20]. A study to reduce alcohol and drug use among AI/AN youth combined motivational interviewing with traditional practices [9] and asked about participants’ perceptions of this combined intervention model. Youth especially noted a high acceptability for the collaborative model that elicited their perspectives [9].

Another study used focus group discussions to gather feedback from AI/AN providers and community members about adapting motivational interviewing and communication for changing substance use behaviors [28]. Community members talked about several traditional ways of communicating for change—for example, focusing on strengths and using positive framing—that aligned well with motivational interviewing principles and techniques. AI/AN providers found the principles of motivational interviewing to be aligned with their cultural framework and experiences [28].

A study in Canada with Aboriginal and non-Aboriginal youth in residential treatment programs for substance use measured initial therapeutic alliance and treatment engagement [7]. They found that reporting a stronger alliance was associated with better engagement with treatment. However, a discrepancy between predominantly white practitioners’ assessments of having a strong positive alliance with all clients and Aboriginal youth’s less favorable assessment of alliance was present. While these studies align with this study’s findings, more research on therapeutic alliance and communication among practitioners and AI/AN clients in opioid use harm reduction and treatment settings is needed. Utilizing peer support specialists with lived experience can address concerns about counselors not having relatable drug use experience.

### Theme 2: flexibility of services

An important identified facilitator centered around flexibility of services. Specifically, participants suggested that increasing availability of immediate medication initiation and flexibility of intake assessment times can reduce barriers to MOUD. These suggestions, among others, were also delineated in the scoping review by Bozinoff et al. [5] who found that longer prescriptions, streamlined referral processes, bridging inpatient and outpatient treatment programs, waiving prescriber and recipient requirements for prescribing and receiving MOUD, among others factors that increased flexibility, increased retention and engagement in MOUD. Implementing flexible service delivery models for MOUD care has been identified as a facilitator to care by others who work in low-resource settings in the US [6]. A systematic review of 38 studies that examined barriers from clients’ perspectives found lack of flexibility to be a major theme across studies [10].

Among AI/AN populations, implementing service delivery models that can flexibly address the needs of a population facing inequities in access to substance use care can provide a community-based approach that more accurately reflects the needs of AI/AN clients. One possibility to enhance flexibility further might be to make intake or follow-up assessments available via telehealth. A study done with Alaska Native clients compared 103 people receiving telepsychiatry with 103 others who were receiving usual care. They found that while those receiving telepsychiatry had higher rates of legal challenges, post-traumatic stress disorder, having children in outside custody, and a history of violence, they were more likely to complete treatment and less likely to discharge from services against provider advice [16]. Future research should explore other creative solutions that will enhance service delivery flexibility and reduce barriers to care.

### Theme 3: social determinants needs as barriers to care

Another core finding was that individual needs rooted in social determinants of health played a significant role in participants’ ability and willingness to seek care. For instance, in this rural area, participants overwhelmingly cited unmet transportation needs. Similar social determinants of health concerns were also highlighted by Bozinoff and colleagues [5] who found that facilitating transportation, verifying that treatment programs and MOUD options were covered by insurance companies, and ensuring basic needs (food, water, housing) were met were associated with higher engagement in MOUD. Having health insurance reimburse clients for transportation costs could mitigate some of the burden related to transportation, especially in rural areas.

### Examples of programs developed by tribal communities to address these barriers/facilitators

Tribal Nations have implemented innovative treatment approaches to increase access to MOUD services [19, 30, 35–37]. Elements of these programs often align with each of the three themes identified in this research to help mitigate barriers to MOUD.

For example, programs developed by Tribal Nations have emphasized connection to others (Theme 1) by offering clinical services with multiple sources of clinical support (e.g., traditional healers, psychiatrists, therapists/counselors, social workers), training tribal members to act as post-overdose peer support specialists, incorporating tribal elders and other community members in the treatment setting (e.g., as greeters), and sharing meals and other culturally meaningful moments of connection. They have focused on addressing individual needs (Theme 2) by coordinating and providing transportation to treatment facilities, providing childcare while patients are in treatment facilities, finding ways to reduce financial burdens associated with getting treatment, offering telephone assistance to support patients remotely when needed, and operating transitional housing and shelters. They have aimed to increase the flexibility of services (Theme 3) by removing punitive sanctions for people who use drugs while in treatment, implementing harm reduction approaches to “meet individuals where they are at,” educating patients, substance use disorder treatment clinicians, and other healthcare workers (e.g., emergency medical services workers) to maximize harm reduction practices and accept harm reduction goals, and fostering relationships with other community groups to reduce stigma associated with OUD and MOUD and to increase acceptance of treatment approaches (MOUD, harm reduction, etc.).

### How Tribal Nation Behavioral Healthcare has changed as a result of the research

This research was conducted in March of 2022, just after the height of the COVID-19 pandemic. The Tribal Nation behavioral health system is characterized by its willingness to try new things to better serve Anishinaabe clients/relatives, and its commitment to creating sustainable models of healthcare. In addition, because of the close CBPR working relationship between the University and Tribal Nation members, in which the Community Action Board and other members of the Tribal Nation weigh in on the research and help steer its direction, the initial research findings were iteratively shared with the CAB, as they unfolded. The CAB Board met with the research team between the first and second week of data collection to hear about the first week of research and inform the next week of questions and data collection. Additional meetings have followed. Thus, some of the barriers described in this paper have been addressed by the Tribal Nation’s Behavioral Healthcare system since the time of the interviews, and some facilitators have been noted and enhanced. This was facilitated by having individuals on the CAB who are in leadership roles in the Tribal Nation (e.g., tribal government, director of services department, clinical leadership roles), and had the ability to influence these changes. This is one method to translate research findings into action.

Since the research study, the Tribal Nation has prioritized hiring more drivers to make transportation easier for MOUD program clients. The number of transportation staff has increased two-to-three times, though there are still some unfilled positions for drivers. Additionally, it is now easier to access and start treatment. Previously, clients had to complete a state-mandated Rule 25 assessment before starting Suboxone. Same day appointments were not always available. Now, clients do not have to wait for a state-mandated intake assessment to begin buprenorphine. This reflects a combination of tribal-level and state-level changes to make treatment access easier. On June 30, 2022, a few months after the research interviews were completed, the state moved from the Rule 25 assessment to a comprehensive assessment. In conjunction with these changes, the Tribal Nation Behavioral Health Division has built programming that prioritizes ease of access. Individuals have the option to complete a same-day medical intake assessment with an intake provider. The consent is all on one page. A more formal comprehensive assessment is still completed but not required immediately. Additionally, peer support staff are able to administer the comprehensive assessment.

Building on the flexibility aspect, clients can also be granted a special request to take home doses of buprenorphine, so they may not need to dose at the clinic every day. Cultural activities are now available almost daily. The clinic also made the reception area more welcoming, including having smudge and other traditional medicines available. Clinic staff have made efforts to destigmatize buprenorphine across the health system (e.g., addressing beliefs that one cannot be “sober” or achieve “recovery” while receiving MOUD). Clinical leaders within the health system continue to explore several other ways to reduce barriers to MOUD for people in the community (e.g., exploring ways to reduce barriers related to mandated assessment requirements, the lack of methadone treatment options within the area).

### Strengths and limitations

This was one of the first studies to ask clients at a Syringe Service Programs about barriers and facilitators to opioid care in a rural, Indigenous setting. There exists ongoing partnerships between tribal members and the university team. This has allowed the involvement of those with lived experience. These individuals led the development of questions and assisted with formulating other protocols for this study. Interviews were facilitated by a non-Native research partner from outside of the community and data analysis was conducted by a diverse team (individuals identifying as AI/AN, white, different age groups and backgrounds, etc.). While it was attempted to avoid bias by gathering multiple perspectives, these factors could have influenced the way clients responded and analyses were carried out. Additionally, the brief length of the interviews and subsequent excerpts included above could be a limiting factor. The average length was eight minutes.

### Conclusion

Participants in this study identified several barriers and facilitators related with three main themes that mapped onto domains of the Aanji’bide Model of Opioid Recovery and Change: connection to others, flexibility of services, and social determinants needs that are barriers to seeking and receiving care. Connection to others focused on connections with family, which most discussed as a facilitator, connection with providers as imperative for wellness, and connection with community. Flexibility of services emerged as an important facilitator, especially as it related to the ability to start MOUD when interested. Several barriers associated with individual needs, particularly transportation, also were important to MOUD treatment-seeking and care. Tailoring services to address identified barriers and leverage facilitators of connection and flexibility will enhance care.

## Supplementary Information

Additional file 1.

## Data Availability

No datasets were generated or analysed during the current study.

## Abbreviations

CAB: Community Action Board
OUD: Opioid use disorder
CBPR: Community-based participatory research
MOUD: Medications for opioid use disorder
AI/AN: American Indian and Alaska Native
