# Supplementary material for: Barriers and facilitators of opioid treatment among Indigenous Syringe Services Program clients
Source: Addict Sci Clin Pract. 2025 Oct 16;20:81. doi: 10.1186/s13722-025-00604-8 (PMC12532990; doi:10.1186/s13722-025-00604-8)
Supplement: Supplementary file 1 — Additional file 1. [file 13722_2025_604_MOESM1_ESM.docx]

**Appendix**

**INDIVIDUAL INTERVIEW GUIDE**

**Version 1 (11/10/21)**

**Introduction**

“Like I said, my name is [NAME], and I am [TITLE]. Thank you for taking part in this interview. I’m going to be asking you five questions about opioid treatment services, mental health, spirituality, and self-care. It will take about 15-30 minutes. There are no right or wrong answers. You don’t have to answer any questions you don’t want to answer. As a reminder, your answers are confidential. I hope you will feel comfortable expressing your thoughts and feelings because I am here to learn from you. Do you have any questions for me before we get started? [ANSWER QUESTIONS.] Ok, let’s begin!”

**Access to Treatment Services Question**

1. What things have made it easier for you to access opioid treatment services?
2. What things have made it more difficult for you to access opioid treatment services?

**Mental Health**

1. Many people who use opioids also live with physical and mental health struggles. Has this been part of your journey? If so, please tell me more about that.

(PROBE: If people only talk about physical OR mental health struggles, ask them about the other if they don’t mention it. For example, “And what about mental health struggles?”)

**Connection to Traditional Ways of Being and Spirituality/Higher Power**

1. People have lots of different cultural and religious beliefs, or none at all. The last question is about these beliefs. You don’t have to answer and wherever you’re at is okay.
   1. What role have traditional Native or religious practices played in your life?
   2. How has your opioid use impacted this?
2. What do you do to take care of yourself?

(PROBE: Mentally? Physically? Emotionally? Do you take time for a bath? A walk? See friends? Read a book? Walk barefoot on the earth? Spend time in the woods/nature?)
